# Effects of Low Protein Diet on Production Performance and Intestinal Microbial Composition in Pigs

**DOI:** 10.3390/vetsci10110655

**Published:** 2023-11-14

**Authors:** Dong Wang, Guoshun Chen, Wenzhong Li, Mingjie Chai, Hua Zhang, Yingyu Su

**Affiliations:** 1 College of Animal Science and Technology, Gansu Agricultural University, Lanzhou 730070, China; wangd@st.gsau.edu.cn (D.W.); liwz@st.gsau.edu.cn (W.L.); zhanghua1294521686@163.com (H.Z.); 2 Pingliang Animal Husbandry and Fishery Station, Pingliang 744000, China; 18893329597@163.com; 3 College of Animal Science and Technology, Xinjiang Agricultural Vocational Technical College, Changji 831100, China; 18894311207@163.com

**Keywords:** Hexi pig, dietary protein level, production performance, intestinal microflora

## Abstract

**Simple Summary:**

Low protein feed alleviates the shortage of protein feed and improves the production performance of livestock and poultry by reducing the protein content in the feed and adding synthetic amino acids. In this research, by balancing the proportion of four essential amino acids in the feed, the amount of protein feed raw materials was gradually reduced to prepare low protein feed with different levels of crude protein. The impact of this feed on the production performance and gut microbiota of Hexi pigs was evaluated. Low protein feed has improved the slaughter performance and meat quality of pigs and also has an improved effect on the structure of gut microbiota.

**Abstract:**

In order to study the effects of a low protein diet on the production performance and intestinal microbiota composition of Hexi pigs, twenty-seven Hexi pigs with an initial body weight of 60.50 ± 2.50 kg were randomly divided into three groups (control group (CG), group 1 (G1), and group 2 (G2)) and participated in a 60-day finishing trial. The CG was fed a normal protein level diet with a protein level of 16.0%, and G1 and G2 were fed a low protein level diet with protein levels of 14.0% and 12.0%, respectively. The results showed that the low protein level diet had no significant effect on the production performance of Hexi pigs, compared with the CG, the slaughter rate of G1 and G2 increased by 2.49% (*p* > 0.05) and 6.18% (*p* > 0.05), the shear force decreased by 2.43% (*p* > 0.05) and 15.57% (*p* > 0.05), the cooking loss decreased by 24.02% (*p* < 0.05) and 21.09% (*p* > 0.05), and the cooking percentage increased by 13.20% (*p* > 0.05) and 11.59% (*p* > 0.05). From 45 min to 24 h and 48 h after slaughter, each group of pH decreased by 1.02, 0.66, and 0.42. For muscle flesh color, the lightness (L) increased by 13.31% (*p* > 0.05) and 18.01% (*p* > 0.05) in G1 and G2 and the yellowness (b) increased by 7.72% (*p* > 0.05) and 13.06% (*p* > 0.05). A low protein level diet can improve the intestinal flora richness and diversity of growing and finishing pigs. In the jejunum, the ACE index (899.95), Simpson index (0.90), and Shannon (4.75) index were higher in G1 than in the other groups, but the Chao1 index (949.92) was higher in G2 than in the remaining two groups. Proteobacteria, Actinobacteria, Euryarchaeota, and Verrucomicrobia were significantly higher in G1 than in the CG. The relative abundances of *Lactobacillus*, *Terrisporobacter*, and *Megasphaera* in G1 was significantly higher than in the CG (*p* < 0.05). In the cecum, the ACE index (900.93), Chao1 index (879.10), Simpson index (0.94), and Shannon (5.70) index were higher in G1 than in the remaining groups. The Spirochaetes in G2 were significantly higher than in the other groups, but the Verrucomicrobia was significantly lower than in the other groups. The relative abundances of *Lactobacillus* were higher in G1 and G2 than in the CG (*p* > 0.05). The relative abundances of unidentified_Clostridiales and *Terrisporobacter* in G2 were significantly lower than in the CG (*p* < 0.05). The relative abundance of Turicibacter in G1 was significantly lower than in the CG (*p* < 0.05). The relative abundances of other bacterial genera in G1 and G2 were increased by 30.81% (*p* > 0.05) and 17.98% (*p* > 0.05).

## 1. Introduction

At present, the intensification degree of pig breeding is increasing continuously, and the shortage of protein feed raw material and raising costs have become an important problem that restricts the healthy development of the pig breeding industry [1]. With the development of a safe and healthy breeding industry, a low protein diet has gradually attracted the attention of the breeding industry [2]. Under the economic benefits of reducing feeding costs, a low protein diet can save protein resources, improve protein utilization rates, and protect the environment [3,4]. When the amino acid content in pig feed meets the growth needs of animals, it can reduce the proportion of protein feed in feed, without a significant impact on animal growth, but has a significant impact on reducing nitrogen emissions in feces and urine [5,6]. Low protein feed can increase muscle a value, backfat thickness, and loin eye area, improving meat quality [7,8].

The gut microbiota coexists with the host and plays an important role in regulating digestion, intestinal development, nutrient absorption, and metabolism [9]. However, previous studies have shown that many factors such as animal species, sex, age, and diet structure can affect microbial diversity, among which diet change is one of the most important factors [10]. Protein feed is the source of energy and amino acids in animal bodies; it provides a substrate for intestinal microbial fermentation [11] and influences the composition of the animal intestinal microbial community [12]. The basic nutrients for the life activities of gut microbiota are proteins and their metabolites in feed, which maintain various physiological functions of the host [13,14,15]. If dietary protein exceeds the requirement, the stable state of intestinal flora will be disrupted, leading to intestinal disturbance, a waste of nitrogen resources, and environmental pollution [16,17,18]. Yu et al. [19] found that the content of crude protein in feed had no significant impact on the composition of gut microbiota, but a low protein diet significantly affects the composition of cecal digesta of finishing pigs [20], and a high protein diet can increase the health risk of pig colon [21]. Reducing dietary protein can improve the intestinal microbiota composition, and moderately reducing protein levels can improve the bacterial structure of the ileum and colon in adults. Microorganisms that improve intestinal growth have a positive effect on the intestinal health of Bamei pigs [22].

With the national strategy of protection and utilization of germplasm resources, it is urgent to protect and utilize local pig breeds. Hexi pig is a local pig breed in the Hexi Corridor of Gansu Province, with bright red meat and an excellent taste [23]. Early research has shown that with the reduction of protein in feed and the supplementation of four essential amino acids, the production performance and meat flavor of fattening pigs are improved [24]. Therefore, this experiment takes Hexi pigs as the experimental object to explore the effects of different protein levels of feed on their production performance and intestinal microbial composition.

## 2. Materials and Methods

### 2.1. Experimental Design, Animals, and Diets

The pigs were selected from Liuhe Ecological Agriculture and Animal Husbandry Farm (Gaotai County, Gansu Province). The experiment was conducted on 27 Hexi pigs, which were 120 days old and had a similar health status, with an initial body weight of 60.50 ± 2.50 kg. The pigs were randomly assigned to three different protein levels of diet treatment groups. The three feed treatment groups were the control group (CG), group 1 (G1), and group 2 (G2), with 3 replicates per group (3 pigs per replicate). The pre-test period was 7 days, and the trial period was 65 days. The preparation of the diet accorded to the “Compound Feed for Piglets, Growing and Finishing Pigs” standard of low protein diets for growing and finishing pigs and previous research [24]. The pre-test period was 7 days, and the trial period was 60 days. The pigs had free access to feed and water throughout the whole experiment. The composition and nutritional level of the diet are shown in Table 1.

### 2.2. Animal Performance Indicators

Individual weighing was carried out on the 1st and 65th day, and feed consumption was recorded throughout the experiment. The growth performance evaluation indexes included initial weight (IW), final weight (FW), average daily gain (ADG), average daily feed intake (ADFI), and feed-to-weight ratio (F/G).

One pig was randomly selected from the replicates of each treatment group to be weighed and slaughtered. Slaughtering and sampling were strictly carried out in accordance with the “Good Manufacturing Practice for Livestock and Poultry Slaughtering- Pigs” (Chinese Standard GB/T 19479-2019) [25]. After slaughter, the warm carcass was weighed, and the slaughter rate was represented by the ratio of carcass weight to live weight. A total of 5g contents were aseptically collected from the cecum and jejunum of each slaughtered pig and placed in a cryopreservation tube, and quickly placed in liquid nitrogen for subsequent 16S detection.

The average thicknesses of backfat, shear force, and meat color were measured according to the method of Wang et al. [24]. The loin eye area and carcass length were determined according to the method of Wu [26] and Li et al. [27]. Cooking loss and pH were determined according to the method of Skrlep et al. [28].

### 2.3. Intestinal Microbial Composition Analysis

The high-throughput sequencing method was used to detect the microbial abundance of the contents of the jejunum and cecum, and the sequencing was completed by Nuovo Zhiyuan Bioinformatics Technology Co., Ltd. (Beijing, China). DNA was extracted from the intestinal contents; Illumina HiSeq 2500 sequencing was used to perform paired-end sequencing of DNA fragments from V3–V4 communities, and the DADA2 method was used for primer removal, quality filtering, denoising, splicing, and chimerism removal. The specific primers for the variable region of bacterial 16S rRNA V3-V4 were 338F: 5′-ACTCCTACGGGAGGCAGCA-3′ and 806R: 5′-GGACTACHVGGGTWTCTAAT-3′ [29,30]. The analysis steps were as followed:: first, call qiimecutadapt trim-paired to cut off the primer fragments of the sequence and discard the sequences that do not match the primers; then, call DADA2 through qiime dada2 denoise-paired for quality control, denoising, splicing, and chimerism removal [31,32].

### 2.4. Data Processing and Analysis

All data are presented as means ± SE. The data analysis was conducted using one-way ANOVA with IBM SPSS 22.0 Statistics software. Group differences were compared using Duncan’s multiple comparisons. *p* < 0.05 was considered statistically significant.

## 3. Results

### 3.1. Effect of Dietary Protein Level on the Growth Performance of Hexi Pigs

As shown in Table 2, compared with the CG, the FW and ADG of G1 increased by 1.41% (*p* > 0.05) and 3.61% (*p* > 0.05), and the F/G decreased by 5.22% (*p* > 0.05), while the G2 group increased by 13.04% (*p* > 0.05). The ADFI in G2 (3.20 kg·day^−1^) was higher than in the other two groups. Under the conditions of this experiment, reducing feed protein levels did not have any adverse effects on the production performance of Hexi pigs, with a 14.0% protein group showing a trend towards improving the production performance.

### 3.2. Effects of Dietary Protein Levels on Production Performance and Meat Quality of Hexi Pigs

Table 3 shows that compared with the CG, the slaughter rates of G1 and G2 increased by 2.49% (*p* > 0.05) and 6.18% (*p* > 0.05). The carcass lengths increased by 2.85% (*p* > 0.05) and 1.87% (*p* > 0.05), the shear forces decreased by 2.43% (*p* > 0.05) and 15.57% (*p* > 0.05), the cooking losses decreased by 24.02% (*p* < 0.05) and 21.09% (*p* > 0.05), and the cooking percentages increased by 13.20% (*p* > 0.05) and 11.59% (*p* > 0.05), respectively. The backfat thickness of CG was the largest, while that of G1 was significantly lower than that of the other two groups (*p* < 0.05). The loin eye areas of G1 and G2 were significantly lower than that of the CG (*p* < 0.05). In terms of drip loss, G1 was significantly higher than the other two groups (*p* < 0.05); the filtration rate increased, but it did not reach a significant level. From 45 min to 24 h and then 48 h after slaughter, the pH of each group decreased by 1.02, 0.66, and 0.42, respectively. For muscle flesh colors, the lightness (L~* value) increased by 13.31% (*p* > 0.05) and 18.01% (*p* > 0.05) in G1 and G2, the yellowness (b~* value) increased by 7.72% (*p* > 0.05) and 13.06% (*p* > 0.05), and the redness (a~* value) decreased by 3.86% (*p* > 0.05) and 9.54% (*p* > 0.05). Therefore, the low protein level diet had a positive impact on the production performance of Hexi pigs; it can improve the slaughter rate, carcass length, muscle brightness (L* value), and yellowness (b* value), reduce muscle shear force and cooking loss, delay the decline in muscle acidity, and have a positive effect on improving meat quality.

### 3.3. 16S rRNA Sequencing of the Jejunum and Cecum of Fattening Pigs under Different Dietary Protein Levels

**Figure 1:**
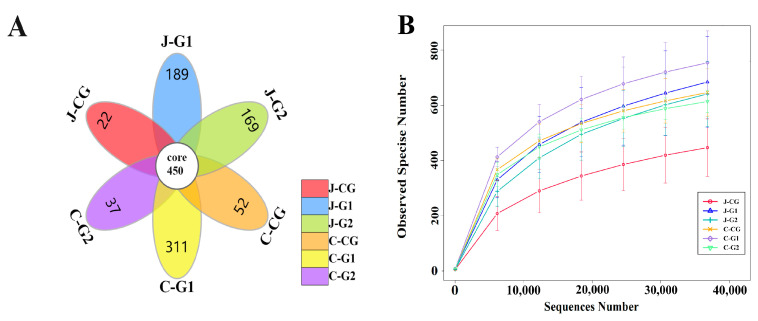
(**A**) OTU petal diagram of jejunum and cecum contents in groups with different protein levels. (**B**) OTU dilution curves of jejunum and cecum contents in groups with different protein levels. J-CG is the content of the jejunum of the CG, J-G1 is the content of the jejunum of G1, and J-G2 is the content of the jejunum of group G2; C-CG is the content of the cecum of the CG, C-G1 is the content of the cecum of G1, and C-G2 is the content of the cecum of G2. These designations also apply to the following figure.

Based on the Illumina Miseq high-throughput sequencing platform, the 16S rRNA gene V3-V4 regions of the jejunal and cecal contents samples were sequenced. After removing incorrect chimeric sequences, the effective data volume for quality control was 59,857, and the effective rate for quality control was 66.41%. A total of 2031 OTUs were identified at the species level using the 99% sequence similarity standard, and the Silva 132 database was used to annotate taxonomic information for each OTU sequence. Figure 1A shows the number of common and unique OTUs in each group of samples. The total number of OTUs in the jejunum and cecum of each group was 450. The numbers of unique OTUs in the jejunum content of the CG, G1, and G2 were 22, 189, and 169, respectively. The numbers of unique OTUs in the CG, G1, and G2 in the cecal contents were 52, 311, and 37, respectively. The curve was flat at 35,000 reads, indicating that the sequencing coverage was saturated (Figure 1B); these data meet the criteria for subsequent result analysis.

### 3.4. Analysis of the Microbial Diversity in the Jejunum and Cecum of Fattening Pigs under Different Dietary Protein Levels

Alpha diversity refers to the diversity within a specific region or ecosystem. As shown in Table 4, in the jejunum, compared to the CG, the ACE indices of G1 and G2 increased by 48.64% (*p* > 0.05) and 43.83% (*p* > 0.05), the Chao1 indices increased by 55.72% (*p* > 0.05) and 62.40% (*p* > 0.05), the Simpson indices increased by 18.42% (*p* > 0.05) and 5.26% (*p* > 0.05), and the Shannon indices increased by 50.79% (*p* < 0.05) and 19.68% (*p* > 0.05). In the cecum, compared to the CG, G1 and G2 Simpson indices increased by 3.30% (*p* > 0.05) and 2.20% (*p* > 0.05), while Shannon indices increased by 8.57% (*p* < 0.05) and 4.00% (*p* > 0.05). The ACE index and Chao1 index of G1 were higher than those of the other groups. The ACE index and Chao1 index of G2 were lower than those of the other groups. Low protein diets can increase the diversity of gut microbiota, but in the cecum, with a decrease in dietary protein levels, microbial diversity first increases and then decreases, with the microbial diversity of G1 being the most significant.

### 3.5. Analysis of the Microbial Composition of the Jejunum and Cecum of Fattening Pigs under Different Dietary Protein Levels

**Figure 2:**
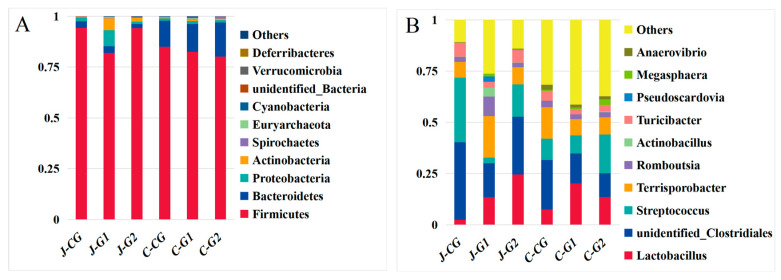
(**A**) Average relative abundances of major bacterial phyla of the jejunum and cecum in Hexi pigs at different protein levels. (**B**) Average relative abundances of major bacterial genera in the jejunum and cecum of Hexi pigs at different protein levels.

Firmicutes, Bacteroidetes, and Proteobacteria were the main predominant phyla of the jejunum and cecum of fattening pigs, as these phyla accounted for more than 95.0% of the total flora (Figure 2A; Supplementary Materials, Table S1). In the jejunum, the proportions of Firmicutes and Bacteroidetes in the CG, G1, and G2 were 28.82, 25.14, and 45.92. The next most abundant phyla were Actinobacteria, Spirochaetes, Euryarchaeota, and Cyanobacteria. Compared with the CG, the relative abundance of Firmicutes in G1 significantly decreased by 13.03% (*p* < 0.05), and the relative abundance of Proteobacteria significantly increased by 332.06% (*p* < 0.05), whereas the relative abundance of Proteobacteria in G2 was significantly lower than in the CG (*p* > 0.05). The relative abundance of the phylum Gastroarchaea was significantly increased in G1 relative to the CG (*p* < 0.05); the relative abundance of Gastroarchaea was also elevated in G2 relative to the CG (*p* > 0.05). The relative abundances of other bacterial phyla in G1 and G2 were significantly increased relative to the CG (*p* < 0.05). In the cecum, the relative abundances of Firmicutes in G1 and G2 were reduced by 2.92% (*p* > 0.05) and 5.75% (*p* > 0.05). The relative abundances of Bacteroides in G1 and G2 increased by 7.20% (*p* > 0.05) and 31.14% (*p* > 0.05). Dietary protein levels could affect the diversity and abundance of jejunum and cecum microorganisms in finishing pigs at the phylum level.

The dominant bacteria in the jejunum contents of the CG were unidentified_Clostriales, Streptococcus, *Terrisporobacter*, and Turicibacter (Figure 2B and Supplementary Materials, Table S2). In the jejunum, *Lactobacillus*, unidentified_Clostriales, *Terrisporobacter*, and Romboutsia were the dominant bacteria contents of G1, and *Lactobacillus*, unidentified_Clostriales, Streptococcus, and *Terrisporobacter* were the dominant bacteria contents of G2; the abundance of Streptococcus genus in the CG was significantly higher than in other groups (*p* < 0.05); the relative abundances of Terriporobacter, Actinobacillus, Pseudoscardovia, and *Megasphaera* genera in the G1 were significantly higher than in the other groups (*p* < 0.05); and the abundance of *Lactobacillus* genus in G2 was significantly higher than in other groups (*p* < 0.05). In the cecum, the relative abundances of Unidentified_Clostridiales, Terriporobacter, and Turicibacter genera in the CG were significantly higher than those of the other groups (*p* < 0.05); the relative abundance of *Lactobacillus* in G1 was higher than in other groups (*p* > 0.05); the abundance of Actinobacillus species in the G2 was significantly higher than in the other groups (*p* < 0.05); and the relative abundances of other bacterial genera in G1 and G2 were increased by 30.81% (*p* > 0.05) and 17.98% (*p* > 0.05), respectively, compared with the CG.

### 3.6. Gene Function Prediction

**Figure 3:**
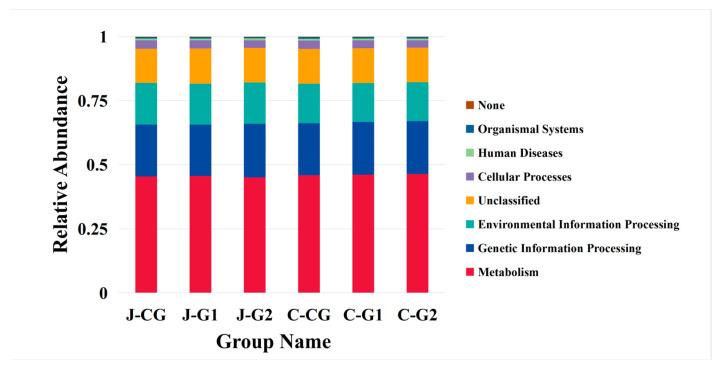
KEGG pathways.

The functional genes of microorganisms in the jejunum and cecum of Hexi pigs were enriched in 10 main KEGG pathways under different protein levels of diet, as shown in Figure 3. Most of the genes were enriched in pathways for metabolism. The difference in gene functional pathways between jejunum and cecal microbiota was mainly manifested in differences in metabolic pathways (Supplementary Materials, Figure S1). There was no significant difference in the KEGG pathway of jejunal microbiota genes. On the contrary, there were significant differences in the KEGG gene enrichment pathway among groups in the cecum (*p* < 0.05), which were mainly manifested by significant differences between the CG and G2 in the human disease pathway (*p* < 0.05). At different dietary protein levels, the annotation abundance of other gene pathways is basically the same and there is no significant difference.

## 4. Discussion

### 4.1. Effect of the Dietary Protein Level on the Growth Performance of Hexi Pigs

The decrease in protein levels in feed has no significant effect on the growth performance of finishing pigs. Research has found that dietary protein has decreased by 2.0% to 3.0% at normal levels, and adding essential amino acids to meet the nutritional needs of pigs or achieve ideal amino acid ratios has no significant impact on the growth performance of fattening pigs [33,34]. Research has shown that reducing protein in feed and appropriately adding amino acids does not affect the growth performance of fattening pigs [35]. Adding 0.49% alanine and 1% tyrosine to the feed with a crude protein content of 12.52% has a positive effect on the growth performance of finishing pigs [36]. Previous studies have found that low protein diets can significantly improve the gut microbiota composition of Bamei pigs, which has a positive impact on their healthy production [22]. Based on the results of this research, a decrease of 2.0~4.0% in the protein levels of the diet did not significantly affect the growth performance of fattening pigs. In addition, based on the feed prices at the time, the cost of raising fattened pigs was reduced to improve production efficiency.

### 4.2. The Effect of the Dietary Protein Level on the Production Performance and Meat Quality of Hexi Pigs

The results of this experiment indicate that compared to the CG, low protein diets do not affect the carcass traits of finishing pigs. Many reports have varying opinions on the impact of low protein diets on pig carcass traits. Xie et al. [37] and Zhou et al. [38] found that reducing dietary protein levels by three percentage points did not affect the growth performance of finishing pigs; this was consistent with the research of Norgaard et al. [39] and Qin et al. [40]. However, some studies found that reducing dietary protein levels can affect the carcass weight and lean meat percentage of pigs, and the carcass fat content of fattening pigs significantly increases [41]. In this experiment, the ADG of G1 was higher than that of the other groups, indicating an improvement in low protein feed. The magnitude of shear force reflects the tenderness and taste of muscles. Drip loss, cooking loss, and filtration rate mainly reflect the processing quality of pork. The larger the loss value, the more juice loss in the pork, and the poorer the meat quality. Research has found that low protein diets significantly reduce the shear force of muscle in fattening pigs [42]. In this research, low protein diets reduced shear force and cooking loss and increased filtration rate. In the research on meat quality, the degree of pH value reduction can affect the muscle glycogen content, thereby affecting meat quality. Research has found that low protein has no significant effect on muscle color and pH [43,44,45,46,47], but in this experiment, low protein feed showed a trend of increasing muscle L value and b value, while decreasing a value. This research shows that, compared to the CG, with a decrease in dietary protein levels, the slaughter rate and carcass characteristics of G1 and G2 fattening pigs increase, muscle shear force and cooking loss decrease, and meat color increases, slowing down the decrease in muscle pH, indicating that reducing dietary protein has no negative impact on the meat quality of fattening pigs.

### 4.3. The Effect of the Dietary Protein Level on the Diversity and Abundance of Intestinal Flora in Hexi Pigs

Intestinal microbes play a crucial role in the host’s body, regulating the host’s metabolism and gene expression and contributing significantly to intestinal immunity and health [48]. Research has shown a close relationship between nutrition and intestinal flora [49,50]. Diets rich in protein exhibit a higher buffering capacity, resulting in an increased pH value in the small intestine. Consequently, this environment favors the proliferation of pathogenic bacteria [51]. Research by Konstantinov et al. [52] highlights the importance of intestinal microbiota diversity in maintaining stability. Greater microbial diversity is believed to enhance the microbiota’s stability, promoting overall gut health and animal growth. In microecology, Chao1 and ACE are used to estimate species richness, while Shannon and Simpson are used to estimate species diversity. The higher the Shannon index, the higher the diversity of the community species. Research has shown that reducing dietary protein levels can increase the abundance and diversity of ileal microbiota, which is similar to the results of this research [53]. In this research, the α diversity was higher in G1 and G2 than in the CG, and the Shannon index of G1 was significantly higher than that of the other groups, indicating that the diversity of microbial communities in the jejunum of G1 was the highest; similar results were also observed in the cecum. In the cecum, the ACE, Chao1, Simpson, and Shannon indices of G1 were higher than those of the other two groups, indicating the highest species diversity of the gut microbiota community. When the body consumes excessive protein, it can reduce the diversity of gut microbiota and affect health [54,55,56], which is similar to the results of this research.

### 4.4. The Effect of the Dietary Protein Level on the Composition of Intestinal Flora and KEGG Function of Hexi Pigs

By analyzing the relative abundance of fecal microbiota in finishing pigs at the phylum and genus levels, this study found that a low protein diet can affect the composition of fecal microbiota in the intestinal tract of finishing pigs. Many studies have reported that Firmicutes and Bacteroidetes can participate in the glycolysis process, can promote energy absorption and metabolism in the body, and are the dominant phyla in pig gut microbiota [57,58]. The microbial composition in the intestine will shift towards more beneficial bacterial species as the protein in the diet decreases, with the most obvious being bacteria used for fermenting carbohydrates [59]. At the phylum level, this research also obtained the same results. The dominance of the main phyla of fecal microbiota in the three groups of fattening pigs was ranked from highest to lowest, namely Firmicutes, Bacteroidetes, and Proteobacteria. This indicates that Firmicutes and Bacteroidetes occupy an absolute advantage in the gut microbiota, and reducing dietary protein levels does not alter the structure of the main gut microbiota in fattening pigs. Research has found that when the crude protein level in the diet decreases, the relative abundance of Firmicutes in the ileum of finishing pigs decreases, while the relative abundance of Proteobacteria increases [60]. In the relative abundance of jejunal microorganisms, the relative abundance of Firmicutes in G1 was significantly lower than in the other two groups, and Proteobacteria, Actinobacteria, and Euryarchaeota were significantly higher than in the other two groups; Cyanobacteria was significantly higher in G2 than that in other groups. In the caecum, the relative abundance of Spirochaetes in G2 was significantly higher than in the other groups. The relative abundance of Verrucomicrobia was significantly lower than in the other groups. At the genus level, *Lactobacillus* belongs to Firmicutes. Lactic acid bacteria are a kind of beneficial microorganism that can decompose and transform nutrients such as proteins and carbohydrates into lactic acid and antibacterial substances. They mainly reduce intestinal pH and inhibit the proliferation of potential intestinal pathogens such as Escherichia coli, so as to promote animal health [61]. Research has found that reducing dietary protein levels can increase the number of protein- and amino acid-degrading bacteria as well as butyric acid-producing bacteria [62], which is consistent with the results of this research. In the jejunum, the relative abundance of *Lactobacillus* in G2 was significantly higher than that in other groups; the relative abundance of Streptococcus in the CG was significantly higher than in the other groups; and the relative abundances of Terriporobacter, Actinobacillus, and Pseudoscardovia in G1 were significantly higher than in the other two groups. In the cecum, the relative abundance of *Lactobacillus* in G1 was higher than that in other groups; The relative abundance of Terriporobacter in CG was significantly higher than in the other groups; the relative abundances of Terriporobacter and Actinobacillus in G2 were significantly higher than in the other two groups. Research has shown that reducing protein levels in the diet can improve the metabolic function of gut microbiota [63,64]. This study used PICRUSt to predict the enrichment of functional pathways in each group at the first level based on the KEGG database. It was found that microbial genes in the jejunum and cecum are mainly enriched in metabolic pathways, with the functional pathways of G1 related to environmental information processing and genetic information processing in the jejunum being lower than in the other groups. The cellular processes pathway of G2 was lower than that of the other two groups. In the cecum, the metabolism pathways of the C1 and G2 are higher than that of the CG. It is speculated that low protein diets may promote the metabolic activity of gut microbiota in the cecum.

## 5. Conclusions

This study investigated the effects of feed with different protein levels on the production performance and gut microbiota composition of Hexi pigs. The results showed that a decrease in feed protein levels had no negative impact on the growth performance of Hexi pigs; but it had a positive impact on slaughter performance, meat quality, and gut microbiota.

## Figures and Tables

**Table 1:** Composition and nutrient levels of the basal diet (air-dry basis) [24].

Basic Diet Composition (%)			
Raw Material	CG	G1	G2
Corn	63.50	68.00	72.00
Soybean meal	18.30	12.90	7.20
Wheat bran	5.00	5.00	5.00
Alfalfa meal	5.00	6.50	9.00
Bentonite	4.00	2.90	1.60
Soybean oil	1.50	1.70	2.00
Compound Enzyme preparation	0.10	0.10	0.10
Lys	0.09	0.23	0.37
Met	-	0.03	0.05
Thr	-	0.08	0.15
Try	-	0.03	0.05
CaCO_3_	0.51	0.46	0.30
CaHPO_4_	1.15	1.22	1.33
0.5% fattening Pig core feed ^①^	0.50	0.50	0.50
NaCl	0.35	0.35	0.35
Total	100.00	100.00	100.00
Nutrient levels ^②^			
DE/(MJ/kg)	13.14	13.13	13.11
CP	15.94 (16.00% ^③^)	14.00 (14.00% ^③^)	12.05 (12.00% ^③^)
CF	3.22	3.50	4.02
Ca	0.60	0.61	0.60
TP	0.55	0.55	0.55
Na	0.16	0.16	0.17
Cl	0.27	0.27	0.28
SID Lys	0.86	0.86	0.86
SID Met	0.26	0.26	0.26
SID Thr	0.59	0.59	0.59
SID Try	0.19	0.19	0.19

^①^ The premix provided the following per kg of diets: Fe 64.00 mg, Zn 71.00 mg, Mn 35.00 mg, Cu 17.00 mg, Se 0.36 mg, I 0.64 mg, Vitamin A 790 IU, Vitamin D3 135 IU, Vitamin E 55.00 mg, thiamine (Vitamin B1) 2.20 mg, riboflavin (Vitamin B2) 2.50 mg, biotin 0.05 mg, folic acid 0.35 mg, nicotinic acid 29.00 mg, calcium pantothenate 27.00 mg, Vitamin B6 0.09 mg, Vitamin B12 1.00 mg, choline chloride 5000.00 mg, flavoring agent 3000.00 mg, sweetening agent 3000.00 mg, phytase 4000.00 mg, Lys 30,000.00 mg, and Try 2000.00 mg. ^②^ Nutrient levels were all calculated values. ^③^ Test set values are in brackets.

**Table 2:** Effect of dietary protein level on the growth performance of Hexi pigs.

Items	CG	G1	G2	*p*-Value
Initial body weight/kg (IW)	61.68 ± 2.16	61.45 ± 1.95	61.58 ± 2.19	0.788
Final body weight/kg (FW)	111.48 ± 3.26	113.05 ± 2.81	110.78 ± 3.22	0.498
Average daily feed intake/kg·day^−1^ (ADFI)	3.02 ± 0.15	2.97 ± 0.16	3.20 ± 0.13	0.387
Average daily gain/kg·day^−1^ (ADG)	0.83 ± 0.03	0.86 ± 0.05	0.82 ± 0.06	0.169
Ratio of feed to gain(F/G)	3.64 ± 0.06	3.45 ± 0.11	3.90 ± 0.15	0.125

**Table 3:** Effects of dietary protein levels on production performance and meat quality of Hexi pigs.

Items	CG	G1	G2	*p*-Value
Slaughter rate/%	67.99 ± 0.02	69.68 ± 0.33	72.19 ± 2.96	0.055
Backfat thickness/mm	36.85 ± 5.72 ^ab^	23.80 ± 4.31 ^b^	31.83 ± 11.13 ^a^	0.006
Carcass length/cm	115.83 ± 4.44	119.13 ± 9.50	118.00 ± 4.58	0.225
Loin eye area/cm^2^	58.53 ± 7.56 ^a^	44.08 ± 6.20 ^ab^	30.75 ± 9.25 ^b^	0.013
Drip loss/%	5.45 ± 1.15 ^b^	7.53 ± 0.69 ^a^	4.82 ± 1.74 ^b^	0.005
Cooking loss/%	35.46 ± 4.07 ^a^	26.94 ± 7.53 ^b^	27.98 ± 8.33 ^ab^	0.005
Cooking percentage/%	64.54 ± 2.97	73.06 ± 8.56	72.02 ± 9.29	0.402
Filtration rate/%	6.34 ± 1.07	7.11 ± 1.38	7.21 ± 7.12	0.126
Shear force/N	44.50 ± 7.14	43.42 ± 6.69	37.57 ± 5.27	0.614
pH_45min_	6.23 ± 0.23 ^a^	5.88 ± 0.16 ^b^	6.03 ± 0.33 ^ab^	0.023
pH_24h_	5.30 ± 0.06 ^b^	5.34 ± 0.04 ^b^	5.66 ± 0.31 ^a^	<0.01
pH_48h_	5.21 ± 0.09 ^b^	5.22 ± 0.15 ^b^	5.61 ± 0.26 ^a^	0.002
L_45min_	34.42 ± 2.80	39.00 ± 5.95	40.62 ± 5.09	0.097
a_45min_	13.73 ± 2.48	13.20 ± 1.93	12.42 ± 1.66	0.522
b_45min_	7.12 ± 1.32	7.67 ± 1.73	8.05 ± 1.16	0.508

a,b Means with distinct superscripts within the same row indicate significant differences (*p* < 0.05).

**Table 4:** Effects of dietary protein levels on the α diversity of the intestinal microbiota of Hexi pigs.

Items	CG	G1	G2	*p*-Value
Jejunum				
ACE	605.44 ± 148.78	899.95 ± 280.22	870.83 ± 214.62	0.275
Chao1	584.91 ± 141.34	910.85 ± 295.45	949.92 ± 376.38	0.306
Simpson	0.76 ± 0.03	0.90 ± 0.06	0.80 ± 0.07	0.058
Shannon	3.15 ± 0.56 ^b^	4.75 ± 0.46 ^a^	3.77 ± 0.65 ^ab^	0.035
Cecum				
ACE	795.33 ± 120.47	900.93 ± 173.35	731.85 ± 58.40	0.324
Chao1	798.85 ± 120.28	879.10 ± 147.68	723.18 ± 42.19	0.309
Simpson	0.91 ± 0.01	0.94 ± 0.02	0.93 ± 0.03	0.430
Shannon	5.25 ± 0.11	5.70 ± 0.40	5.46 ± 0.59	0.459

a,b Means with distinct superscripts within the same row indicate significant differences (*p* < 0.05).

## Data Availability

The original data presented in the study are included in the article, see Section 3; further inquiries can be directed to the corresponding author.

## Supplementary Materials

The following supporting information can be downloaded at: https://www.mdpi.com/article/10.3390/vetsci10110655/s1, Table S1. Relative abundance on phylum level; Table S2. Relation abundance on genus level; Figure S1. Differential gene function pathways.

## References

[1] Oliveira C.H., Bernardes R.D., Dias K.M.M., Ribeiro A.M., Rodrigueiro R.J.B., Koo B.K., Tak J., Park C., Calderano A.A., Albino L.F.T. (2023). Research Note: The influence of different isoleucine: Lysine ratios on the growth performance of broiler chickens fed low-protein diets. Poult. Sci..

[2] Rocha G.C., Duarte M.E., Kim S.W. (2022). Advances, Implications, and Limitations of Low-Crude-Protein Diets in Pig Production. Animals.

[3] Wang Y., Han S., Zhou J., Li P., Wang G., Yu H., Cai S., Zeng X., Johnston L.J., Levesque C.L. (2020). Effects of dietary crude protein level and N-carbamylglutamate supplementation on nutrient digestibility and digestive enzyme activity of jejunum in growing pigs. J. Anim. Sci..

[4] Fu R., Liang C., Chen D., Tian G., Zheng P., He J., Yu J., Mao X., Gu Z., Yang W. (2023). Effects of low-energy diet supplemented with betaine on growth performance, nutrient digestibility, and serum metabolomic profiles in growing pigs. J. Anim. Sci..

[5] Gloaguen M., Le F.N., Corrent E., Primot Y., Milgen J. (2014). The use of free amino acids allows formulating very low crude protein diets for piglets. J. Anim. Sci..

[6] Wang B., Mi M.M., Zhang Q.Y., Bao N., Pan L., Zhao Y., Qin G.X. (2021). Relationship between the amino acid release kinetics of feed proteins and nitrogen balance in finishing pigs. Animal.

[7] Xu Y., Chen H., Wan K., Zhou K., Wang Y., Li J., Tang Z., Sun W., Wu L., An R. (2022). Effects of supplementing low-protein diets with sodium dichloroacetate and glucose on growth performance, carcass traits, and meat quality of growing-finishing pigs. J. Anim. Sci..

[8] Shriver J.A., Carter S.D., Sutton A.L., Richert B.T., Senne B.W., Pettey L.A. (2003). Effects of adding fiber sources to reduced-crude protein, amino acid-supplemented diets on nitrogen excretion, growth performance, and carcass traits of finishing pigs. J. Anim. Sci..

[9] Isaacson R., Kim H.B. (2012). The intestinal microbiome of the pig. Anim. Health Res. Rev..

[10] Li F., Li C., Chen Y., Liu J., Zhang C., Irving B., Fitzsimmons C., Plastow G., Guan L.L. (2019). Host genetics influence the rumen microbiota and heritable rumen microbial features associate with feed efficiency in cattle. Microbiome.

[11] Ma N., Tian Y., Wu Y., Ma X. (2017). Contributions of the Interaction Between Dietary Protein and Gut Microbiota to Intestinal Health. Curr. Protein Pept. Sci..

[12] Bikker P., Dirkzwager A., Fledderus J., Trevisi P., le Huërou-Luron I., Lallès J.P., Awati A. (2006). The effect of dietary protein and fermentable carbohydrates levels on growth performance and intestinal characteristics in newly weaned piglets. J. Anim. Sci..

[13] Abdallah A., Elemba E., Zhong Q., Sun Z. (2020). Gastrointestinal Interaction between Dietary Amino Acids and Gut Microbiota: With Special Emphasis on Host Nutrition. Curr. Protein Pept. Sci..

[14] Davila A.M., Blachier F., Gotteland M., Andriamihaja M., Benetti P.H., Sanz Y., Tomé D. (2013). Intestinal luminal nitrogen metabolism: Role of the gut microbiota and consequences for the host. Pharmacol. Res..

[15] Bishu S. (2016). Sensing of nutrients and microbes in the gut. Curr. Opin. Gastroenterol..

[16] Chen J., Li Y., Tian Y., Huang C., Li D., Zhong Q., Ma X. (2015). Interaction between Microbes and Host Intestinal Health: Modulation by Dietary Nutrients and Gut-Brain-Endocrine-Immune Axis. Curr. Protein Pept. Sci..

[17] Wang Y., Zhou J., Wang G., Cai S., Zeng X., Qiao S. (2018). Advances in low-protein diets for swine. J. Anim. Sci. Biotechnol..

[18] Wu L., Zhang X., Tang Z., Li Y., Li T., Xu Q., Zhen J., Huang F., Yang J., Chen C. (2018). Low-Protein Diets Decrease Porcine Nitrogen Excretion but with Restrictive Effects on Amino Acid Utilization. J. Agric. Food Chem..

[19] Yu D., Zhu W., Hang S. (2019). Effects of low-protein diet on the intestinal morphology, digestive enzyme activity, blood urea nitrogen, and gut microbiota and metabolites in weaned pigs. Arch Anim. Nutr..

[20] Luo Z., Cheng Y., Zhu W. (2015). The effect of low-protein diet on the cecal metabolites and flora of fattening pigs. Anim. Husb. Vet. Med..

[21] Wang H., Shen J., Pi Y., Gao K., Zhu W. (2019). Low-protein diets supplemented with casein hydrolysate favor the microbiota and enhance the mucosal humoral immunity in the colon of pigs. J. Anim. Sci. Biotechnol..

[22] Wang D., Chen G., Song L., Chai M., Wang Y., Shui S., Zhang H., Sha Y., Yao Y. (2020). Effects of Dietary Protein Levels on Bamei Pig Intestinal Colony Compositional Traits. Biomed. Res. Int..

[23] (2015). Local Pig Breed—Hexi Pig. Rural. New Technol..

[24] Wang D., Chen G., Chai M., Shi C., Geng Y., Che Y., Li Y., Liu S., Gao Y., Hou H. (2022). Effects of dietary protein levels on production performance, meat quality and flavor of fattening pigs. Front. Nutr..

[25] (2019). Good Manufacturing Practice for Livestock and Poultry Slaughtering—Pig.

[26] Wu L.Y. (2011). The Effect of Microecological Preparations and Achyranthesbidentata Polysaccharides on Pig Growth Performance and Meat Quality. Master’s Thesis.

[27] Li Y.H., Li F.N., Duan Y.H., Guo Q.P., Wen C.Y., Wang W.L., Huang X.G., Yin Y.L. (2018). Low-protein diet improves meat quality of growing and finishing pigs through changing lipid metabolism, fiber characteristics, and free amino acid profile of the muscle. J. Anim. Sci..

[28] Skrlep M., Poklukar K., Kress K., Vrecl M., Fazarinc G., Batorek Lukac N., Weiler U., Stefanski V., Candek-Potokar M. (2020). Effect of immunocastration and housing conditions on pig carcass and meat quality traits. Transl. Anim. Sci..

[29] Magoc T., Salzberg S.L. (2011). FLASH: Fast length adjustment of short reads to improve genome assemblies. Bioinformatics.

[30] Edgar R.C. (2010). Search and clustering orders of magnitude faster than BLAST. Bioinformatics.

[31] Werner J.J., Zhou D., Caporaso J.G., Knight R., Angenent L.T. (2012). Comparison of Illumina paired-end and single-direction sequencing for microbial 16S rRNA gene amplicon surveys. ISME J..

[32] Langille M.G., Zaneveld J., Caporaso J.G., McDonald D., Knights D., Reyes J.A., Clemente J.C., Burkepile D.E., Vega Thurber R.L., Knight R. (2013). Predictive functional profiling of microbial communities using 16S rRNA marker gene sequences. Nat. Biotechnol..

[33] Tuitoek K., Young L.G., de Lange C.F., Kerr B.J. (1997). The effect of reducing excess dietary amino acids on growing-finishing pig performance: An elevation of the ideal protein concept. J. Anim. Sci..

[34] Prandini A., Sigolo S., Morlacchini M., Grilli E., Fiorentini L. (2013). Microencapsulated lysine and low-protein diets: Effects on performance, carcass characteristics and nitrogen excretion in heavy growing-finishing pigs. J. Anim. Sci..

[35] Zhou J., Wang L., Zhou J., Zeng X., Qiao S. (2021). Effects of using cassava as an amylopectin source in low protein diets on growth performance, nitrogen efficiency, and postprandial changes in plasma glucose and related hormones concentrations of growing pigs. J. Anim. Sci..

[36] Shi B.M., Bai G.D., Xu X., He W., Yang Z., Gao F. (2019). Effects of adding tyrosine to low protein diets on the growth performance and nitrogen metabolism of fattening pigs. J. Northeast. Agric. Univ..

[37] Xie C., Zhang S., Zhang G., Zhang F., Chu L., Qiao S. (2013). Estimation of the optimal ratio of standardized ileal digestible threonine to lysine for finishing barrows fed low crude protein diets. Asian-Australas J. Anim. Sci..

[38] Zhou P., Zhang L., Li J., Luo Y., Zhang B., Xing S., Zhu Y., Sun H., Gao F., Zhou G. (2015). Effects of Dietary Crude Protein Levels and Cysteamine Supplementation on Protein Synthetic and Degradative Signaling in Skeletal Muscle of Finishing Pigs. PLoS ONE.

[39] Norgaard J.V., Hansen M.J., Soumeh E.A., Adamsen A.P.S., Poulsen H.D. (2014). Effect of protein level on performance, nitrogen utilisation and carcass composition in finisher pigs. Acta Agric. Scand. A Anim. Sci..

[40] Qin C., Huang P., Qiu K., Sun W., Xu L., Zhang X., Yin J. (2015). Influences of dietary protein sources and crude protein levels on intracellular free amino acid profile in the longissimus dorsi muscle of finishing gilts. J. Anim. Sci. Biotechnol..

[41] Liu S., Xie J., Fan Z., Ma X., Yin Y. (2023). Effects of low protein diet with a balanced amino acid pattern on growth performance, meat quality and cecal microflora of finishing pigs. J. Sci. Food Agric..

[42] Zhu Y.P., Zhou P., Li J.L., Zhang L., Gao F., Zhou G.H. (2017). Effects of Low Protein Level Diets Supplemented with Essential Amino Acids and Cysteamine on Meat Quality and Related Genes Expression of Growing Pigs. Acta Vet. Et Zootech. Sin..

[43] Zhang S., Chu L., Qiao S., Mao X., Zeng X. (2016). Effects of dietary leucine supplementation in low crude protein diets on performance, nitrogen balance, whole-body protein turnover, carcass characteristics and meat quality of finishing pigs. Anim. Sci. J..

[44] Bidner B.S., Ellis M., Witte D.P., Carr S.N., McKeith F.K. (2004). Influence of dietary lysine level, pre-slaughter fasting, and rendement napole genotype on fresh pork quality. Meat Sci..

[45] Goerl K.F., Eilert S.J., Mandigo R.W., Chen H.Y., Miller P.S. (1995). Pork characteristics as affected by two populations of swine and six crude protein levels. J. Anim. Sci..

[46] Ruusunen M., Partanen K., Pösö R., Puolannea E. (2006). The effect of dietary protein supply on carcass composition, size of organs, muscle properties and meat quality of pigs. Livest. Sci..

[47] Li N., Xie C.Y., Zeng X.F., Wang D.H., Qiao S.Y. (2018). Effects of dietary crude protein level and amino acid balance on growth performance, carcass traits and meat quality of finishing pigs. Chin. J. Anim. Nutr..

[48] Guo X., Xia X., Tang R., Zhou J., Zhao H., Wang K. (2008). Development of a real-time PCR method for Firmicutes and Bacteroidetes in faeces and its application to quantify intestinal population of obese and lean pigs. Lett. Appl. Microbiol..

[49] Valdes A.M., Walter J., Segal E., Spector T.D. (2018). Role of the gut microbiota in nutrition and health. BMJ.

[50] Matsui T., Tanaka J., Namihira T., Shinzato N. (2012). Antibiotics production by an actinomycete isolated from the termite gut. J. Basic Microbiol..

[51] Htoo J.K., Araiza B.A., Sauer W.C., Rademacher M., Zhang Y., Cervantes M., Zijlstra R.T. (2007). Effect of dietary protein content on ileal amino acid digestibility, growth performance, and formation of microbial metabolites in ileal and cecal digesta of early-weaned pigs. J. Anim. Sci..

[52] Konstantinov S.R., Favier C.F., Zhu W.Y., Williams B.A., Klüß J., Souffrant W.B., de Vos W.M., Akkermans A.D., Smidt H. (2005). Microbial diversity studies of the porcine gastrointestinal ecosystem during weaning transition. Anim. Res..

[53] Fan P., Liu P., Song P., Chen X., Ma X. (2017). Moderate dietary protein restriction alters the composition of gut microbiota and improves ileal barrier function in adult pig model. Sci. Rep..

[54] Rist V.T., Weiss E., Eklund M., Mosenthin R. (2013). Impact of dietary protein on microbiota composition and activity in the gastrointestinal tract of piglets in relation to gut health: A review. Animal.

[55] Jensen B.B., Piva A., Knudsen K.E.B., Lindberg J.E. (2001). Possible ways of modifying type and amount of products from microbial fermentation in the gut. Gut Environment of Pigs.

[56] Zhao J., Zhang X., Liu H., Brown M.A., Qiao S. (2019). Dietary Protein and Gut Microbiota Composition and Function. Curr. Protein Pept. Sci..

[57] Greenhill C. (2015). Gut microbiota: Firmicutes and Bacteroidetes involved in insulin resistance by mediating levels of glucagon-like peptide 1. Nat. Rev. Endocrinol..

[58] Shoaie S., Karlsson F., Mardinoglu A., Nookaew I., Bordel S., Nielsen J. (2013). Understanding the interactions between bacteria in the human gut through metabolic modeling. Sci. Rep..

[59] Opapeju F.O., Krause D.O., Payne R.L., Rademacher M., Nyachoti C.M. (2009). Effect of dietary protein level on growth performance, indicators of enteric health, and gastrointestinal microbial ecology of weaned pigs induced with postweaning colibacillosis. J. Anim. Sci..

[60] Fan P. (2016). The Effect of Low-Protein Diets on the Gut Microflora of Weaned Piglets and Fattening Pigs. Master’s Thesis.

[61] Xia Y., Zhang Y., Xu J., Guo S., Ding B. (2019). Effects of *Lactobacillus* fermentum and Bacillus coagulans on growth performance and intestinal health of broilers infected by Clostridium perfringens. China Anim. Husb. Vet. Med..

[62] Wang J., Wang S., Liu H., Zhang W., Zhang D., Wang Y., Ji H. (2018). Effects of low protein level diets on the growth performance and intestinal flora of growing-finishing pigs. J. Anim. Nutr.

[63] Devillard E., McIntosh F.M., Duncan S.H., Wallace R.J. (2007). Metabolism of linoleic acid by human gut bacteria: Different routes for biosynthesis of conjugated linoleic acid. J. Bacteriol..

[64] Fan P., Li L., Rezaei A., Eslamfam S., Che D., Ma X. (2015). Metabolites of Dietary Protein and Peptides by Intestinal Microbes and their Impacts on Gut. Curr. Protein Pept. Sci..

