# Supplementary material for: Effects of Low Protein Diet on Production Performance and Intestinal Microbial Composition in Pigs
Source: Vet Sci. 2023 Nov 14;10(11):655. doi: 10.3390/vetsci10110655 (PMC10675339; doi:10.3390/vetsci10110655)
Supplement: Supplementary file 1 [file vetsci-10-00655-s001.zip › vetsci-2623626-supplementary.pdf]

*Supplementary Materials*

**Table S1.** Relative abundance on phylum level.

| Items | CG | G1 | G2 | *P*-value |
|---|---|---|---|---|
| | | Jejunum | | |
| Firmicutes | 94.24±3.98[a] | 81.96±6.73[b] | 94.13±3.75[a] | 0.038 |
| Bacteroidetes | 3.27±0.88 | 3.26±1.37 | 2.05±0.94 | 0.350 |
| Proteobacteria | 1.84±1.18[b] | 7.95±2.52[a] | 1.23±0.74[b] | 0.005 |
| Actinobacteria | 0.42±0.24[b] | 5.86±1.81[a] | 1.97±1.09[b] | 0.004 |
| Spirochaetes | 0.0199±0.0150 | 0.0706±0.0534 | 0.0751±0.0557 | 0.324 |
| Euryarchaeota | 0.0081±0.0054[b] | 0.5286±0.3467[a] | 0.0253±0.0229[b] | 0.031 |
| Cyanobacteria | 0.0407±0.0272[b] | 0.0425±0.0041[b] | 0.1285±0.0397[a] | 0.013 |
| unidentified_Bacteria | 0.0788±0.0309 | 0.0625±0.0483 | 0.0299±0.0151 | 0.280 |
| Verrucomicrobia | 0.0009±0.0004[b] | 0.0127±0.0062[a] | 0.0091±0.0040[ab] | 0.037 |
| Deferribacteres | 0.0625±0.0211[a] | 0.0018±0.0016[b] | 0.0018±0.0016[b] | 0.001 |
| Others | 0.0217±0.0047[b] | 0.2535±0.0968[a] | 0.3657±0.1012[a] | 0.005 |
| | | Caecum | | |
| Firmicutes | 85.04±7.96 | 82.56±7.29 | 80.15±8.68 | 0.764 |
| Bacteroidetes | 12.78±8.45 | 13.70±8.38 | 16.76±7.56 | 0.827 |
| Proteobacteria | 1.21±0.20 | 1.34±0.40 | 1.23±0.67 | 0.931 |
| Actinobacteria | 0.5341±0.3358 | 0.9097±0.1027 | 0.7088±0.3423 | 0.334 |
| Spirochaetes | 0.1439±0.0322[b] | 0.3286±0.0958[b] | 0.9351±0.2382[a] | 0.002 |
| Euryarchaeota | 0.0045±0.0031 | 0.3657±0.3307 | 0.0525±0.0204 | 0.116 |
| Cyanobacteria | 0.0742±0.0551 | 0.0797±0.0467 | 0.0290±0.0122 | 0.340 |
| unidentified_Bacteria | 0.0326±0.0262 | 0.0127±0.0031 | 0.0063±0.0025 | 0.170 |
| Verrucomicrobia | 0.0507±0.0087[a] | 0.0724±0.0185[a] | 0.0072±0.0057[b] | 0002 |
| Deferribacteres | - | 0.0027±0.0005 | - | 0.000 |
| Others | 0.1249±0.0476[b] | 0.6273±0.2879[a] | 0.1276±0.0667[b] | 0.018 |

a,b Means with distinct superscripts within the same row indicate significant differences ($p < 0.05$).

**Table S2.** Relation abundance on genus level.

| Items | CG | G1 | G2 | *P*-value |
|---|---|---|---|---|
| | | Jejunum | | |
| *Lactobacillus* | 2.457±1.108$^{c}$ | 13.413±3.408$^{b}$ | 24.578±4.754$^{a}$ | 0.001 |
| *unidentified_Clostridiales* | 37.798±1.310 | 16.683±7.263 | 28.236±23.947 | 0.277 |
| *Streptococcus* | 31.612±3.537$^{a}$ | 2.725±1.195$^{c}$ | 15.714±6.939$^{b}$ | 0.001 |
| *Terrisporobacter* | 7.598±3.376$^{b}$ | 20.181±8.206$^{a}$ | 8.425±4.054$^{b}$ | 0.060 |
| *Romboutsia* | 2.446±1.007 | 9.523±8.104 | 2.171±0.835 | 0.179 |
| *Actinobacillus* | 0.122±0.031$^{b}$ | 4.370±1.805$^{a}$ | 0.123±0.075$^{b}$ | 0.021 |
| *Turicibacter* | 6.799±3.031 | 2.877±1.640 | 6.057±4.136 | 0.328 |
| *Pseudoscardovia* | 0.087±0.068$^{b}$ | 2.565±1.133$^{a}$ | 0.128±0.114$^{b}$ | 0.006 |
| *Megasphaera* | 0.139±0.008$^{b}$ | 1.250±0.708$^{a}$ | 0.431±0.361$^{ab}$ | 0.059 |
| *Anaerovibrio* | 0.117±0.046 | 0.118±0.050 | 0.176±0.020 | 0.215 |
| Others | 10.826±8.018$^{b}$ | 26.296±7.494$^{a}$ | 13.962±6.142$^{ab}$ | 0.086 |
| | | Caecum | | |
| *Lactobacillus* | 7.512±7.247 | 20.269±6.672 | 13.578±7.436 | 0.171 |
| *unidentified_Clostridiales* | 24.121±2.674$^{a}$ | 14.558±2.904$^{b}$ | 11.617±3.776$^{b}$ | 0.007 |
| *Streptococcus* | 10.391±3.011 | 8.835±6.464 | 18.914±8.693 | 0.204 |
| *Terrisporobacter* | 15.333±1.350$^{a}$ | 7.908±1.528$^{b}$ | 8.320±1.824$^{b}$ | 0.002 |
| *Romboutsia* | 3.129±1.525 | 2.320±0.555 | 2.437±0.580 | 0.588 |
| *Actinobacillus* | 0.130±0.052$^{b}$ | 0.051±0.016$^{b}$ | 0.457±0.145$^{a}$ | 0.003 |
| *Turicibacter* | 4.663±0.916$^{a}$ | 2.384±1.280$^{b}$ | 3.174±0.704$^{ab}$ | 0.077 |
| *Pseudoscardovia* | 0.073±0.069 | 0.085±0.076 | 0.016±0.008 | 0.379 |
| *Megasphaera* | 0.595±0.254 | 0.854±0.605 | 2.730±2.151 | 0.170 |
| *Anaerovibrio* | 2.470±1.941 | 1.425±1.410 | 1.494±0.471 | 0.622 |
| Others | 31.582±9.335 | 41.312±9.333 | 37.262±12.502 | 0.555 |

a,b Means with distinct superscripts within the same row indicate significant differences ($p < 0.05$).

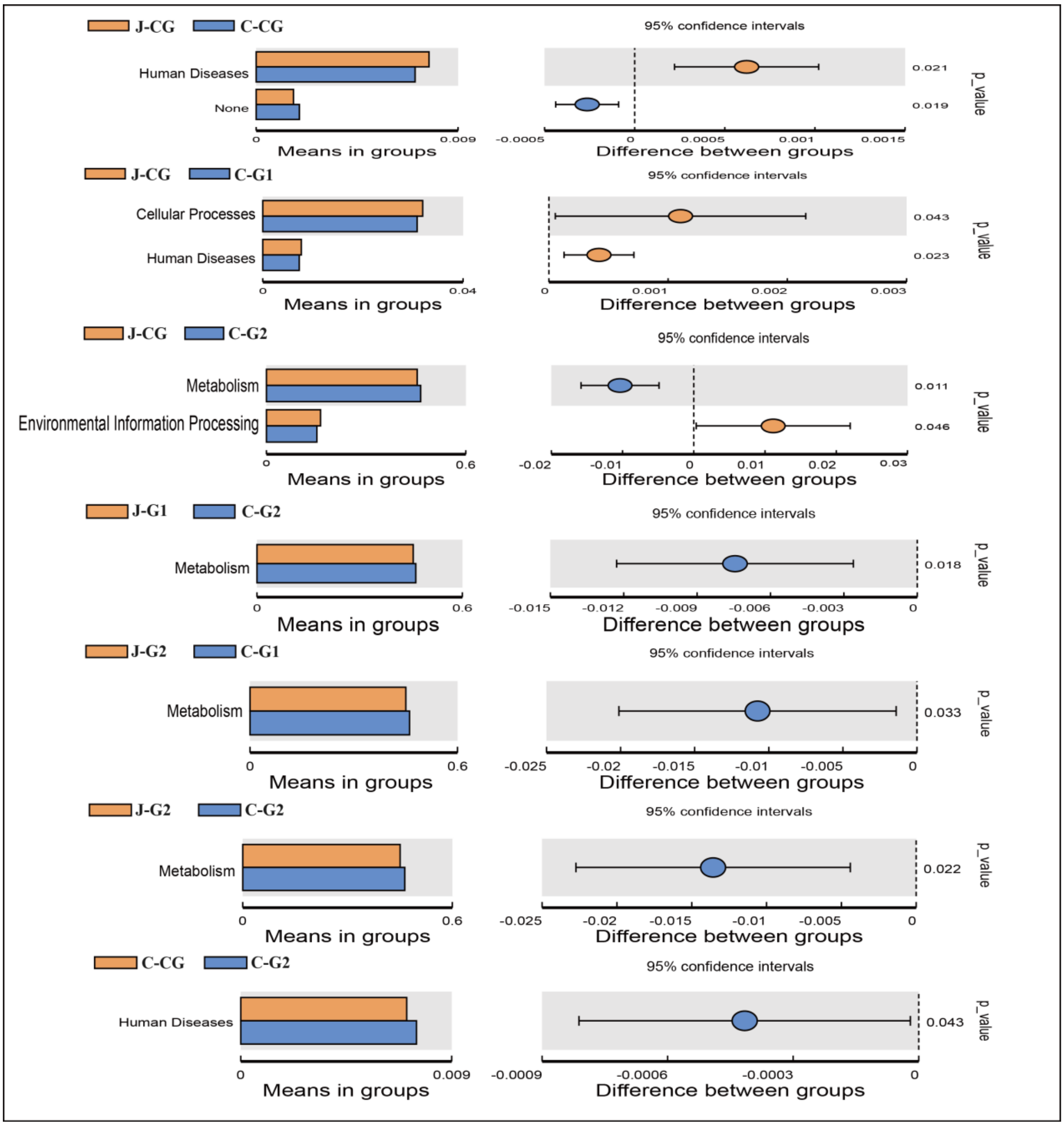


**Figure S1.** Differential gene function pathways.
